# Neurodevelopment as blueprint: biological validation of the dual-architecture AGI framework

**DOI:** 10.3389/frobt.2026.1902952

**Published:** 2026-10-08

**Authors:** Nan Chen

**Affiliations:** Independent Researcher, Chengdu, Sichuan, China

**Keywords:** artificial general intelligence, continual learning, curiosity-driven exploration, dual architecture, neurodevelopment, catastrophic forgetting, token migration

## Abstract

Human intelligence does not develop linearly: it follows a precise phase sequence. This article presents the hypothesis that this neurodevelopmental timeline corresponds to the dual-architecture artificial general intelligence (AGI) system previously proposed by the author, covering the 10 architectural components. We hypothesize that AGI development may be constrained by similar phase sequencing, address key objections (see Sections 3.1, 5.4, 5.5, and 5.6 for explicit objection-response pairs) through formal arguments, and provide indirect supporting evidence for six individual mechanisms on the MuJoCo platform: four-method anti-forgetting comparison: elastic weight consolidation (EWC), synaptic intelligence (SI), memory-aware synapses (MAS), and Naive. Curiosity-driven exploration, training stability analysis, multimodal cross-prediction, and warm-start routing analysis reveal negative transfer in genuinely novel scenarios, and a multi-novelty gradient experiment spanning five gravity levels (1.0×–2.0×) identifies a crossover threshold at approximately 1.25×–1.50× gravity on HalfCheetah-v5. An appendix provides a formal mathematical theory of token migration with two formal sketches and one implication.

## 1 Introduction: why AGI needs developmental biology

The dominant artificial intelligence (AI) paradigm assumes intelligence can be achieved through “larger models + more data.” However, the only known success case produced by 3.5 billion years of evolution, human intelligence, does not operate this way.

Humans are not born thinking. A newborn brain contains approximately 100 billion neurons, but cortical synaptic connections are sparse; executive function is near zero; there is no episodic memory, no language. Yet within 3 years, this system develops causal reasoning, object permanence, basic physical intuition, and a complete training mechanism for continuously absorbing and validating new knowledge.

This article advances a thesis: the human neurodevelopmental timeline is not an accidental biological detail. It is a precisely orchestrated architecture deployment sequence. This sequence corresponds, component by component, to the dual-architecture artificial general intelligence (AGI) design we previously proposed. If this hypothesized correspondence holds, it yields a critical engineering implication: AGI may be fundamentally constrained by developmental stage ordering, which is a testable hypothesis awaiting empirical verification. Machine intelligence may need to retrace key developmental milestones of biological intelligence. The architecture of this thesis is shown in Figure 1.

**FIGURE 1:**
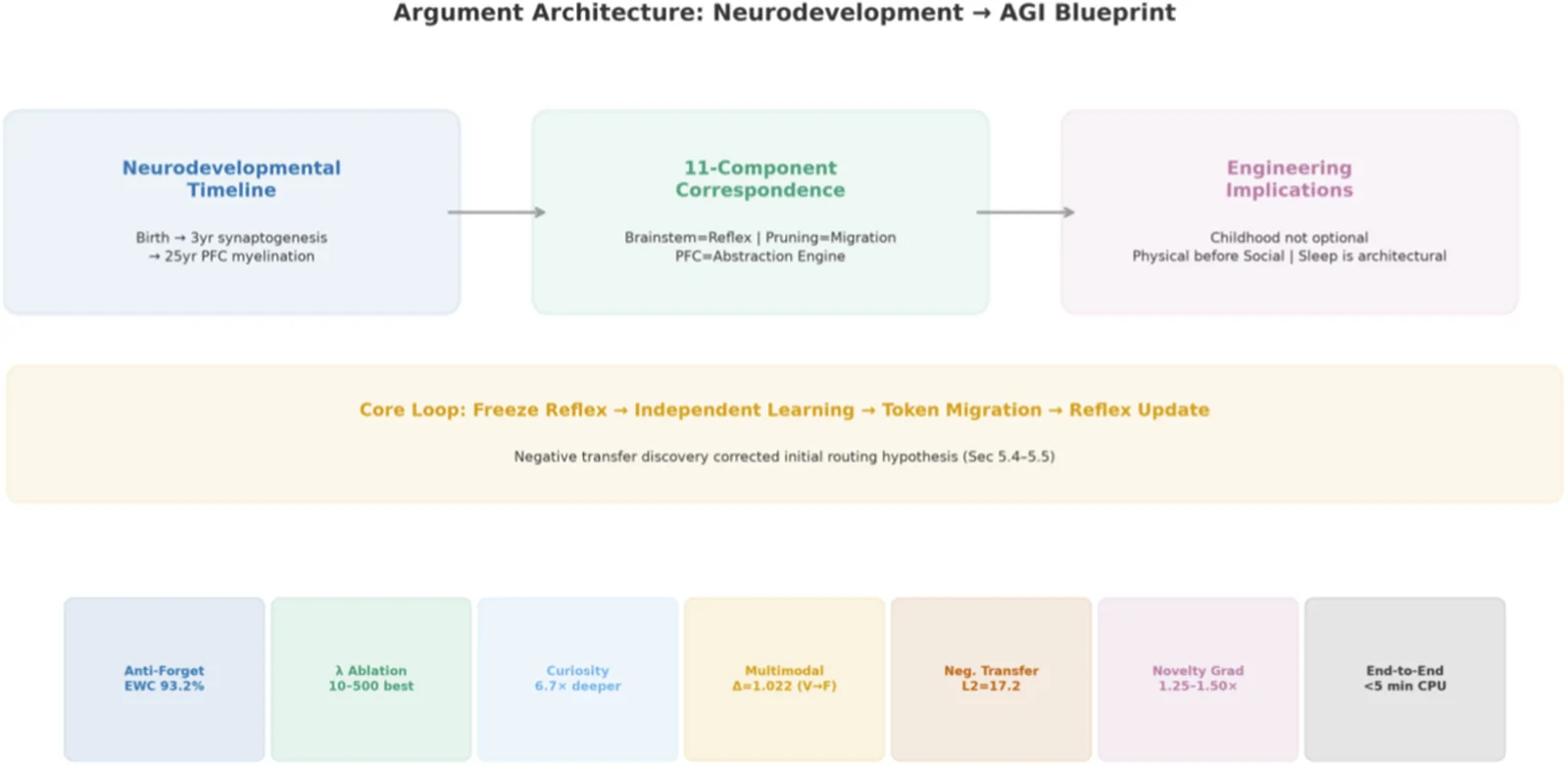
Full argument architecture.

This hypothesis is falsifiable: if an AGI system can be shown to achieve human-level physical and social reasoning without respecting the proposed developmental ordering (physical→social token acquisition sequence), the central claim of this article would be refuted. We explicitly state this as the falsifying condition throughout.

## 2 Related work: developmental perspectives and AGI architecture

This work sits at the intersection of four research domains: developmental neuroscience, continual learning, embodied intelligence, and AGI architecture design. Below, we survey the most directly relevant prior work and clarify this article’s incremental contributions.

1. Developmental neuroscience. Huttenlocher (1979) first quantitatively described the trajectory of human cortical synaptic density, finding that synaptic density at ages 2–3 is approximately double adult levels. Giedd et al. (1999) and Sowell et al. (2003) revealed through longitudinal MRI studies that prefrontal cortical myelination continues until approximately age 25. Yakovlev and Lecours (1967) established the chronological myelination sequence of brain regions. However, these classic findings have not yet been systematically mapped onto AGI architectures. A primary contribution of this article is establishing precise correspondences between this developmental timeline and the 10 components of the dual-architecture AGI system (Section 3).
2. Continual learning and catastrophic forgetting. McCloskey and Cohen (1989) first described catastrophic forgetting in neural networks. Over the past decade, Kirkpatrick et al. (2017) proposed elastic weight consolidation (EWC), Zenke et al. (2017) proposed synaptic intelligence (SI), and Aljundi et al. (2018) proposed memory-aware synapses (MAS). These methods are effective on standard benchmarks (Parisi et al., 2019), but within AGI architecture they are typically treated as plugins: training tricks rather than necessary architectural components. This article takes a different stance: based on developmental correspondences, we argue that continual learning is not an optional training technique but a hard architectural requirement for AGI (the sleep-necessity argument in Section 5.4 and EWC experiments in Section 6.1 both support this position).
3. Embodied intelligence and symbol grounding. Harnad’s (1990) symbol grounding problem remains unsolved. Held and Hein’s (1963) classic kitten experiment demonstrated the necessity of active movement for visual development. More recently, Brooks (1991) and Pfeifer and Bongard (2006) established the theoretical foundations of embodied intelligence. In the AGI domain, LeCun’s (2022) World Model architecture includes perception modules and a world model but does not explicitly incorporate neurodevelopmental temporal sequencing. DeepMind’s embodied multimodal work (e.g., Gato, as reported by Reed et al., 2022) explores multi-task embodied strategies at the engineering level but similarly lacks the concept of developmental stages. This article’s core incremental contribution is transforming the vaguely stated principle of “physical interaction precedes abstract reasoning” into actionable developmental stage ordering and testable experimental predictions.
4. AGI architecture proposals. Several influential AGI architecture proposals have emerged in recent years. Schmidhuber’s (2015) Gödel Machine emphasizes self-referential formal reasoning but neglects developmental ordering. Kurzweil’s (2012) Predictive reverse engineering (PRTM) relies on reverse-engineering the human brain without proposing independent architecture principles. Goertzel’s (2014) OpenCog contains multiple subsystems but lacks developmental stage constraints. More recent work has begun to converge on components this framework predicts: self-supervised predictive world models such as image-based joint embedding predictive architecture (I-JEPA) (Assran et al., 2023) and video-based joint embedding predictive architecture (V-JEPA) (Bardes et al., 2024) pursue latent prediction without hand-crafted labels; RoboCat (Bousmalis et al., 2023) demonstrates a self-improving embodied agent whose capabilities grow through staged experience accumulation. Capability analyses of large language models (Bubeck et al., 2023) document what text-only training can and cannot achieve without physical grounding. None of these lines of work, however, incorporates a developmental ordering constraint as an architectural principle. Compared to these proposals, the dual-architecture system of this article (Chen, 2026) is distinctive in: (a) explicitly encoding developmental timeline as an architectural constraint; physical tokens must precede social tokens; (b) providing neurobiological correspondences for every architectural component; (c) all experiments are reproducible on consumer-grade hardware. We do not claim that the dual architecture is the only viable AGI path, but we do argue that regardless of the chosen architecture, developmental stage ordering is likely unavoidable. The prior work (Chen, 2026) is a non-peer-reviewed preprint; the present article extends and empirically tests its hypotheses. Agreement with the prior architecture should not be interpreted as independent confirmation.

## 3 Three-phase neurodevelopmental timeline

### 3.1 Phase zero: birth; only base code

At birth, the only fully myelinated structures are the brainstem (respiration, heartbeat, basic arousal), spinal reflex arcs (grasp reflex, startle reflex, rooting reflex), and portions of thalamic and basal ganglia circuits. These are “factory pre-installed.” No learning is required; they are operational immediately, with millisecond response times.

In the dual-architecture AGI, this corresponds to the frozen reflex layer: a pretrained vision-language model with fixed weights that delivers predictions in a single forward pass. The infant brainstem enables breathing without “learning” to breathe; the AGI reflex layer enables recognizing “cup” and “table” without learning vision from scratch.

Critical data point: the cortex is almost entirely unmyelinated at birth. The prefrontal cortex, which is the core of human intelligence, is completely unmyelinated. This means the most intelligent part of humans develops after interaction with the environment, not before. This corresponds directly to the AGI design principle that the continuous learning layer’s weights are initialized near-randomly at the start of training.

### 3.2 Phase one: 0–3 years; training mechanisms come online

After birth, the brain launches two massive operations:

First, synaptic overproduction (synaptogenesis). By ages 2–3, cortical neurons average approximately 15,000 synapses, which is twice the adult density. The brain deliberately creates redundant connections, building an enormous hypothesis space, then allows experience to prune it. This is not learning; it is building an architecture capable of learning.

Second, myelination proceeds from posterior to anterior. The visual cortex myelinates within months of birth, followed by the somatosensory cortex, the motor cortex, language areas (temporal lobe), and finally, the prefrontal cortex. This sequence is not random. It maps precisely onto the behavioral developmental sequence: see first, then touch, then move, then speak, and only finally plan and inhibit. Simultaneously, the thalamus and association cortex myelinate during ages 0–2, providing the hardware substrate for multimodal binding. Infants do not separately learn “the seen cup” and “the felt cup;” thalamocortical circuits bind them into a single object token based on temporal synchrony. In the AGI cross-modal translation layer, this corresponds to aligning the outputs of visual and tactile encoders in latent space via contrastive learning: signals from the same moment are pulled together; signals from different moments are pushed apart.

In the AGI architecture, this corresponds to the period when the continuous learning layer is operating at full capacity. Weights are open for updates, and every physical interaction (dropping spoons, pushing blocks, touching hot objects) generates training signals. Two key sub-mechanisms also launch during this phase:

- Dopamine prediction error signaling: When actual outcomes deviate from expectations, midbrain dopamine neurons produce phasic firing (burst activation or suppression). This is not a reward signal; it is a prediction error signal. Schultz et al. (1997) demonstrated that dopamine neurons encode not reward itself but the difference between predicted and actual reward. When predictions are accurate, dopamine does not fire, forming the biological basis for the AGI architecture’s “curiosity module R = KL(P_reflex ||Q_learning) → 0 when predictions align.”
- Hippocampal sleep replay: Wilson and McNaughton (1994) discovered that hippocampal place cell activation sequences from daytime maze running are replayed at approximately 20× speed during REM sleep. This is not random activation. It is the day’s experiences being replayed. Subsequent work (Buzsaki, 2015) demonstrated that this replay is the key mechanism for transferring short-term hippocampal memories to long-term cortical storage. This directly corresponds to the AGI architecture’s replay buffer: sampling old task data and mixing it with new task data during training to prevent forgetting.

### 3.3 Phase two: 3–4 years; the memory model locks

Two simultaneous events occur at ages 3–4, jointly marking the system’s transition from a training phase to a consolidation phase.

First, large-scale synaptic pruning initiates. The brain systematically eliminates unused synaptic connections. The pruning principle is use it or lose it: circuits repeatedly validated by experience are preserved and myelinated; unused circuits are cleared. By the end of adolescence, approximately 50% of childhood synapses are pruned. This is the precise biological counterpart of token migration in the AGI architecture. Tokens verified across N interactions (prediction accuracy > τ) are promoted and injected into the reflex layer for consolidation; unverified candidate patterns are discarded.

Second, infantile amnesia ends. Most humans’ earliest persistent memories form at ages 3–4. Pre-age-3 experiences are not absent. Their storage format (hippocampal-dependence level, cortical representation stability) is inaccessible to the retrieval system that matures later. Josselyn and Frankland (2012) proposed that infantile amnesia results from excessive hippocampal neurogenesis rates: the continuous addition of new neurons overwrites old memory traces. When neurogenesis rates decline around ages 3–4, stable episodic memory formation begins. This has a profound counterpart in the AGI architecture: only after tokens are formally named by the abstraction engine and written into the constraint graph (establishing structural relationships with other tokens) does the experience become retrievable knowledge. Before that, it exists only implicitly as weight updates.

A key engineering insight from this developmental timeline: the “memory lock” at ages 3–4 is not a sudden event. It occurs when synaptic pruning, hippocampal neurogenesis deceleration, and prefrontal myelination initiation simultaneously reach critical thresholds. AGI token migration may similarly require “multi-condition simultaneous satisfaction” triggers, which are precisely the three migration conditions defined in our revised architecture (accuracy > τ, EWC importance < η, minimum interaction count > K_min).

We can provide a concrete example of token migration from “force” to “persistence:” A robot in Phase 1 learns the physical token “force” through 1,000 box-pushing interactions: its vector encoding v_force captures the causal structure “push → object accelerates,” and its symbolic rule is “IF push(obj, direction) THEN obj.velocity[direction] + = f/mass.” In Phase 3, while observing human arguments (social interaction), the robot notices a pattern: “one party repeatedly expressing the same stance → the other party eventually shifts behavior.” The abstraction engine recognizes structural similarity with “force:” sustained directional input eventually changes the target state. The engine applies a vector transformation to v_force (via a lightweight multi-layer perceptron (MLP) learning the mapping from physical to social domains), aligning the transformed vector with the candidate social token “persistence;” that is, minimize ||W·v_force − v_persistence||^2^, where W is a learnable domain-transfer matrix. Upon verification, “persistence” receives an independent symbolic rule (“IF repeat(stance, N) THEN partner.stance shifts”), but its vector encoding subspace is initialized from v_force via W. This is not “force = persistence” as a metaphor; mathematically, it is transfer learning: the causal structure of the physical domain is reused as a representational prior for the social domain.

### 3.4 Phase three: 3–25 years; the long myelination of the prefrontal cortex

The prefrontal cortex, which is responsible for executive function, planning, impulse inhibition, and theory of mind, begins myelination around age 3 and continues until approximately age 25 (Giedd et al., 1999; Sowell et al., 2003). This is the last region in the entire brain to complete myelination.

Why does the most advanced function complete last? Because social cognition is built on top of physical cognition. You cannot understand the social token “promise” until you have acquired, through physical interaction, the intuition that “an action constrains future behavior,” which first requires understanding that gravity constrains object motion, friction constrains sliding, and collision constrains penetration.

Recent work on self-referential social cognition further reinforces this developmental ordering (Markose, 2024). Self-reference, which is the capacity to model oneself as an entity within one’s own world model, requires prior calibration of sensorimotor, social, and conceptual tokens before self-modeling can be grounded. An agent that cannot predict the physical consequences of its actions cannot meaningfully represent “self” as a causal entity. In human development, self-recognition emerges only after basic sensorimotor coordination is established (mirror self-recognition typically emerges at 18–24 months). In the dual-architecture framework, self-reference corresponds to a special class of token, the self-token, which must be composed from lower-level physical and social tokens, consistent with the principle that higher cognitive functions are built upon, and constrained by, developmental precursors.

This provides powerful neurodevelopmental support for the AGI architecture’s requirement that social tokens must be acquired after physical tokens. The prefrontal cortex cannot myelinate first, not because biology cannot do it, but because prefrontal circuits depend on inputs from sensory cortex, motor cortex, and limbic system to perform planning and inhibition. If those inputs have not been calibrated by physical experience, prefrontal planning is ungrounded, just as an AGI attempting social token discovery before physical token discovery would float in a space of text symbols without anchors.

## 4 Neurodevelopment ↔ AGI architecture: proposed correspondence table

**Table:** 

AGI component	Neurobiological counterpart	Developmental timeline	Key references
Frozen reflex layer	Brainstem/spinal cord/basal ganglia	Complete at birth	—
Continuous learning layer	Cortical synaptic plasticity	Peak 0–3 years	Huttenlocher, 1979
Cross-modal translation	Thalamus + association cortex	Myelination 0–2 years	Yakovlev and Lecours, 1967
Physical feedback signal	Sensorimotor closed loop	Continuous from birth	Held and Hein, 1963
Curiosity R = KL(P||Q)	Dopamine prediction error	Active from birth	Schultz et al., 1997
Replay buffer	Hippocampal REM replay	From ∼1 year	Wilson and McNaughton, 1994
Token migration (τ threshold)	Synaptic pruning + myelination	Accelerates from age 3	Giedd et al., 1999
Token naming/constraint graph	End of infantile amnesia	Age 3–4	Josselyn and Frankland, 2012
Delayed social token acquisition	Prefrontal myelination	Age 3–25	Sowell et al., 2003
Safety constraint layer	Amygdala fear conditioning	Basic at birth, ongoing	LeDoux, 2000

Key reference entries are illustrative anchors from the developmental neuroscience literature, not direct empirical support for each specific AGI-side claim in the correspondence table.

### 4.1 Isomorphism of physical and computational constraints

Objection 1 (Substrate): The brain’s developmental timeline is determined by biophysical constraints (energy metabolism, cranial volume, molecular signaling speed), not computational optimality. How do you prove this is necessary for silicon-based AGI?

Our response: Although carbon and silicon substrates differ physically, both face computational optimization objectives with deep structural isomorphism.

- Myelination ↔ Model compression and inference latency optimization. Biological myelination increases signal conduction speed (from ∼1 m/s unmyelinated to ∼100 m/s myelinated) and reduces energy consumption. In AGI, this precisely corresponds to migrating frequently used tokens from the continuous learning layer (requiring gradient updates and carrying a high inference cost) to the reflex layer (single forward pass, low inference cost). This fast, automatic pathway versus the slow, effortful one parallels the distinction between intuitive and deliberate modes of thought in human cognition (Kahneman, 2011). Both systems share the same optimization objective: high-frequency computational paths should be hardened to reduce latency and energy cost.
- Synaptic Pruning ↔ Network Pruning and Generalization. Substantial experimental evidence in artificial neural networks shows that models first trained with over-parameterization, then pruned, achieve better generalization than directly trained compact models. Frankle and Carbin (2019) demonstrated the lottery ticket hypothesis: large randomly initialized networks contain sparse subnetworks that can be independently trained to match the full network’s performance, suggesting over-parameterization + pruning may not be wasteful but necessary for discovering optimal substructures. In the biological brain, the childhood synaptic density of 200% of adult levels may represent the same principle implemented in carbon.
- Sensorimotor Calibration Before Abstract Reasoning ↔ Hierarchical Dependency in Transfer Learning. The prefrontal cortex cannot myelinate before the sensory cortex because prefrontal inputs come from the sensory cortex. If the sensory cortex is not yet calibrated by the physical world, prefrontal planning operates in an ungrounded feature space, which is precisely analogous to how training a high-level classifier on unconverged low-level features in deep learning causes performance collapse. The biological necessity of developmental sequence may simply be the mathematical necessity of hierarchical learning systems, expressed in carbon.

In other words: the brain develops this way not because it is made of flesh but because it is a hierarchical learning system that must bootstrap abstract concepts from raw data. Silicon-based AGI faces the same bootstrapping problem, so the natural engineering solution may also require a similar hierarchical sequence. This is not imitation; it is both systems being constrained by the same mathematical structure.

## 5 Engineering implications for AGI development

### 5.1 Developmental stages cannot be skipped

If the correspondence argued in this article holds, then an AGI system cannot skip the developmental sequence of physical token discovery → migration → social token discovery. Attempting to train social reasoning directly in large language models (LLMs) (e.g., “please analyze the trust dynamics in this conversation”) while bypassing physical interaction has no biological precedent. Humans can perform social reasoning because they first learned that cups fall, falls hurt, and promises can be broken. Symbols must be grounded in the physical world (Harnad, 1990), and grounding takes time; not computational time, but developmental time.

### 5.2 Childhood is not a bug; it is a feature

The synaptic overproduction → pruning sequence suggests a counterintuitive engineering principle: to obtain an efficient final model, you must first build an excessively redundant intermediate model and then use experience to prune it. This appears to be wasteful: why create twice the synapses only to cut half? Biology suggests this may not be waste but the only viable path: only by first possessing a sufficiently large hypothesis space (redundant synapses) can experience effectively select the optimal compressed representation. Training a compact model directly may trap it in local minima, whereas the overproduce → prune path finds better global solutions. For AGI architecture, this means that the continuous learning layer may initially require far larger capacity than ultimately needed; token migration is not merely consolidating knowledge but progressively compressing the model.

### 5.3 Physical → social token sequence is a hardware constraint

The reason that the prefrontal cortex cannot myelinate first is not that social cognition is more difficult; it is because prefrontal inputs come from the sensory cortex and limbic system. If those inputs have not been calibrated by the physical world, the prefrontal cortex processes ungrounded signals. This holds equally for AGI: if the abstraction engine discovers the candidate token “promise” during social interaction, but “promise’s” vector representation depends on the concept of constraint, and constraint must first be discovered from the physical world (gravity constraining objects), then the sequence is mandatory. It is not a case of “it is better to do physical before social” but rather, “doing social presupposes that physical is already internalized in the reflex layer.”

### 5.4 The engineering necessity of sleep

Objection 2 (Biological Accident): Hippocampal replay occurs during REM sleep. This is not a biological accident but likely a necessary offline consolidation phase for any continual learning system. Without sleep (training pause + replay buffer reactivation), the continuous learning layer would continuously overwrite old weights during online updates (catastrophic forgetting). Sleep provides two engineering-essential conditions: (a) suspending new data input, halting weight updates, and (b) concentrated replay of old data during the pause for consolidation. AGI systems may require explicitly implemented sleep cycles, not as a metaphor, but as an architecture-level design that divides training time into online interaction phases and offline consolidation phases.

### 5.5 The timescale paradox: clock time vs. experience time

Objection 3 (Time Scale): The prefrontal cortex takes 25 years to fully develop. Must AGI also train for 25 years? This is engineering unacceptable.

Response: This objection conflates clock time with experience time. In a human, 25 years is clock time limited by carbon-based metabolism: neuronal signal conduction at ∼100 m/s, physical interaction at real-world second timescales, sleep occupying only 8 h per day. AGI faces none of these constraints: physics simulators can run at 100× real-time speed; replay buffers can parallel-replay thousands of experience trajectories; training requires no sleep and can run 24/7.

We can conceptualize this with a simple formula: T_development(AGI) = N_required/R_interaction, where N_required is the equivalent number of interactions needed to reach the migration threshold (same as humans), and R_interaction is the AGI’s interaction rate (potentially orders of magnitude higher). If humans need approximately 10^8^ effective physical interactions to build complete physical common sense (a rough estimate based on critical period experience volume for visual system development), and AGI simulation environments can achieve 10^4^ interactions/hour (simulator acceleration + parallelization), then the equivalent development time is approximately 10^4^ h, or approximately 1.1 years. Respecting the developmental sequence does not mean respecting clock time.

Crucially, experience density itself may be constrained by developmental stage. A 3-year-old child needs 3 years, not because they are slow, but because their brain must acquire experiences in a specific order. The visual cortex must myelinate first; then the somatosensory cortex can effectively integrate visuo-tactile information; then the motor cortex can plan actions based on stable perception. If it were possible to cram all these experiences into an infant over the course of 1 week, it is likely that the brain development sequence would not accelerate at all because the myelination of a later stage depends on the completion signals of previous stages. AGI may face the same constraint: you cannot accelerate social token acquisition before physical token migration completes, not because of insufficient compute but because the vector representation of social tokens depends on the vector representation of physical tokens as priors.

### 5.6 A developmental critique of the Transformer architecture

Objection 4 (Scaling): Current mainstream LLMs are based on pure Transformer architectures. Some might object, “LLMs have already demonstrated reasoning abilities through scaling laws. Why take the detour of infant development?”

From the developmental perspective of this article, Transformers have three architecture-level deficiencies:

- No independent memory buffer. All Transformer memory is encoded in weights and key-value (KV) caches. No structurally independent hippocampal equivalent for experience replay and memory consolidation exists. This makes catastrophic forgetting a fundamental obstacle to continual learning. Although parameter-efficient fine-tuning (PEFT) methods such as low-rank adaptation (LoRA) can mitigate forgetting, they do not provide an independent offline consolidation phase, which is precisely the engineering essence of hippocampus–cortex dialogue.
- No explicit planning module. Transformer self-attention is a form of implicit distributed reasoning. No structurally identifiable prefrontal equivalent for conscious sequential planning and hypothesis testing exists. This means that when the model needs to think before acting, it must complete all reasoning in a single forward pass, with no iterative verification and correction developmental phase.
- No physical grounding mechanism. All Transformer knowledge comes from statistical co-occurrence in text. It “knows” cups fall because it has read related sentences, not because it has experienced the force feedback of releasing an object. The symbol grounding problem (Harnad, 1990) is unsolvable in pure Transformer architectures, not because Transformers are not large enough, but because they have no interface for physical interaction.

This does not mean Transformers are useless for AGI. In contrast, in our dual architecture, the frozen reflex layer can and should be a pretrained Transformer. The problem is not the tool; it is using only one tool. The developmental perspective tells us that a complete intelligence system requires multiple subsystems with different learning dynamics and time scales working in concert. Transformers excel at pattern completion and rapid prediction (reflex layer) but must be coupled with a system capable of incremental online learning (continuous learning layer) and a system capable of causal abstraction and structural transfer (abstraction engine).

### 5.7 Multimodal alignment: from synchrony to unified representations

How does an infant bind “the seen red ball” and “the felt smooth surface” into a single object representation? Developmental neuroscience’s answer is temporal synchrony. When visual and tactile signals are tightly coupled in time (e.g., the moment a hand touches the ball coincides with seeing the ball’s reflective change), the thalamus and association cortex strengthen connections between neurons representing these two modalities via Hebbian learning: neurons that fire together wire together.

In the AGI cross-modal translation layer, this mechanism is implemented through contrastive learning: triplets of (visual frame, force reading, audio clip) from the same moment are trained to be close in embedding space; triplets from different moments are pushed apart. Specifically, the loss function is InfoNCE: L = −log[exp(sim(z_vis, z_force)/T)/Σ exp(sim(z_vis, z_neg)/T)], where T is a temperature parameter, and z_neg represents temporally mismatched negative samples.

A key engineering insight: this alignment is not translation between modalities. It is not converting force signals into visual signals. It maps all modalities into a shared, abstract representational space that transcends any single modality. In this space, the vector representation of “collision” is neither visual nor tactile. It is a conceptual node jointly defined by these modalities under temporal synchrony. Once established, this node can be activated from vision alone (seeing a collision occur → predicting accompanying force and sound), which is precisely the underlying mechanism of the reflex layer’s prediction function.

The deployment path after alignment follows the same pattern as the token migration mechanism described in Section 2.3: verify → freeze → inject into reflex layer. Specifically, (1) in the continuous learning layer, the vision, force, and audio encoders are trained via contrastive learning to achieve temporal alignment in latent space. (2) Once alignment is verified (cross-modal prediction accuracy exceeds threshold), the weights of all three encoders are frozen. (3) The frozen multimodal encoders are injected as a whole into the reflex layer, becoming an intrinsic translation component. Thereafter, the reflex layer, upon receiving any single modality input (e.g., vision), can query the predictions of the other two modalities (force and audio) in latent space because they are already aligned in the shared space. This completes the migration from “the cross-modal translation layer is an independent training module” to “the cross-modal translation layer is an intrinsic component of the reflex layer.”

Optimal routing decision: reflex layer vs. learning layer. After alignment, the core engineering question is, “should each input be routed to the reflex layer or the learning layer?” The experiments in Section 6.4 revealed a critical finding: for sufficiently novel environments, fine-tuning pretrained weights results in negative transfer. The frozen reflex (*R* ^2^ = 0.358) outperforms any fine-tuning variant. Accordingly, the optimal strategy is revised to “frozen reflex + independent learning + confidence-based switching:” (1) All inputs default to the frozen reflex layer, producing multimodal predictions at minimal latency. (2) The system computes prediction confidence. If the forward cross-modal prediction *R* ^2^ exceeds the trust threshold, the reflex output is used directly. (3) If *R* ^2^ falls below the threshold (novel physical patterns), the learning layer is activated but trained independently from random initialization (not by fine-tuning reflex weights); routing switches when the learning layer’s prediction confidence surpasses that of the reflex layer. (4) The reflex layer remains frozen (at high domain novelty) or warm-started (at low domain novelty, per the crossover threshold identified in Section 6.5), and the learning layer is trained independently from random initialization. The two subsystems share no weights and interact only through routing decisions and token migration at the architectural level. This avoids the negative transfer trap when domain discrepancy is large and aligns with the biological fact that reflex arcs do not change through experience. This is the core engineering insight of the dual-architecture design: the reflex layer provides an eternal baseline of immediate response, while the learning layer provides progressive adaptive capability, each performing its own role without interference.

## 6 Indirect supporting evidence for individual mechanisms

To examine the core mechanisms proposed in this article, including anti-forgetting in continual learning, curiosity-driven exploration, training stability, multimodal alignment, reflex-layer routing, and novelty gradient, we implemented a series of verification experiments on the MuJoCo physics engine. All experiments were completed entirely on CPU on a standard MacBook (Intel Core i5 1.6 GHz, no GPU).

### 6.1 EWC anti-forgetting validation

To verify whether the “continuous learning layer + EWC” mechanism can actually prevent catastrophic forgetting, we designed a three-task sequential learning experiment.

Experimental setup: Using the Ant-v5 quadruped robot environment in the MuJoCo physics engine, we designed three sequential learning tasks: Task A (standard gravity, flat terrain), Task B (1.5× gravity, simulating slope training), and Task C (0.3× friction coefficient, simulating ice training). Each task was trained for 300 K steps. To comprehensively evaluate anti-forgetting, we compared four methods: (a) Naive: direct continued training without any protection; (b) EWC: elastic weight consolidation, measuring weight importance via a Fisher information matrix and applying parameter pullback; (c) SI: synaptic intelligence, accumulating gradient information to track per-parameter contributions; (d) MAS: memory-aware synapses, measuring importance via output sensitivity. All methods started from the same pretrained Task A model, were sequentially trained on Tasks B and C, then returned to Task A for retention evaluation.

The results are shown in Figure 2. The Task A baseline score was 878 ± 239. After two sequential task switches (B→C), the four methods achieved: EWC 93.2%, SI 102.3%, MAS 82.7% (all three exceeding the 80% target threshold), and Naive 113.9% (rebounding above baseline, likely due to positive transfer. Exposure to varied dynamics in Tasks B and C produced a policy more robust to standard conditions than the original Task-A-only policy) (positive transfer). After a single task switch (B), EWC achieved 102.5%, SI 108.2%, MAS 67.4%, and Naive 72.2%. EWC was consistently stable, SI showed the strongest single-transfer effect after implementation correction, and MAS improved steadily after the second task switch. All three regularization methods were effective. We note that the Task A baseline coefficient of variation (27%) limits the precision of method comparisons; all percentage differences should be interpreted relative to this inherent variance. Where feasible, future work should employ significance testing (t-test or ANOVA) with correction for multiple comparisons.

**FIGURE 2:**
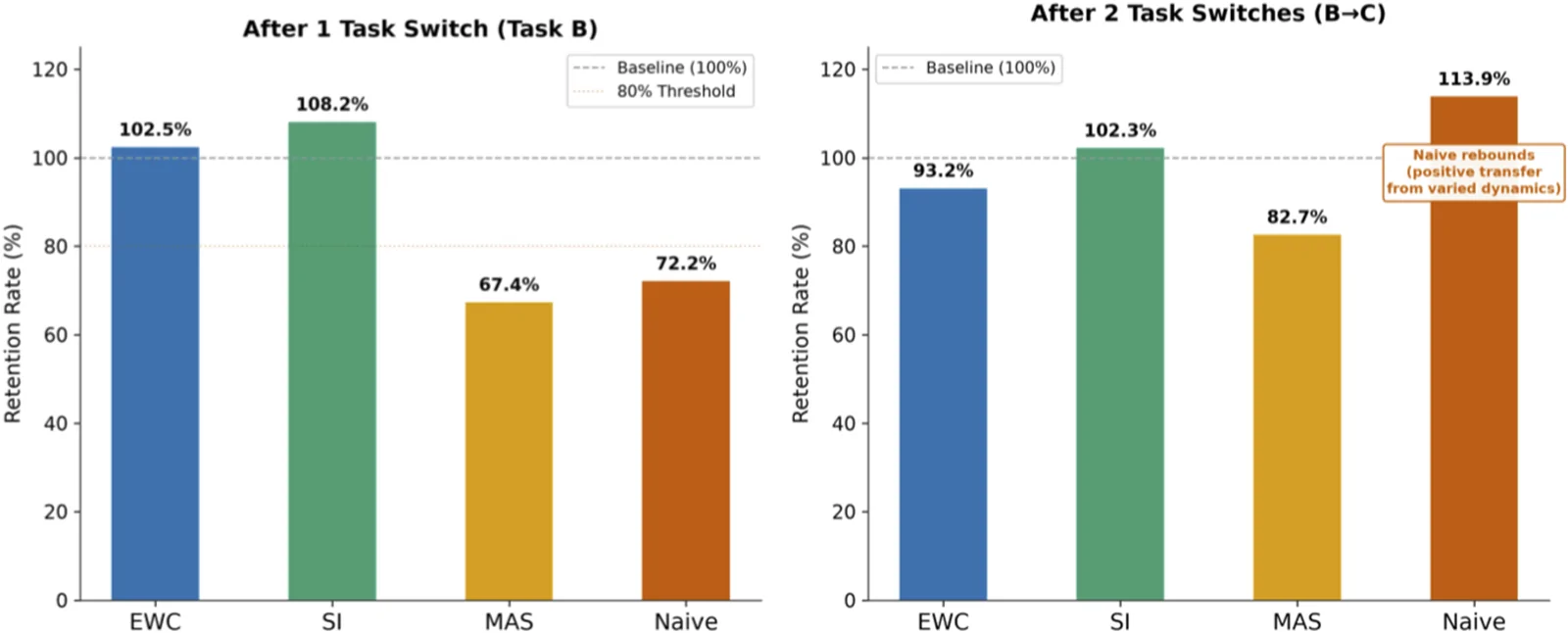
Comparison of four anti-forgetting methods. (Top) Retention rates for EWC, Naive, SI, and MAS after two task switches; (Bottom) Detailed EWC vs. No-EWC comparison with experimental summary.

We further note three implications of the high baseline variance (CV = 27%) for interpreting these results. First, the Naive retention of 113.9%, which exceeds all three regularized methods, may not represent a reliable advantage. With a Task A baseline of 878 ± 239, a single-standard-deviation fluctuation in the baseline can shift the reported retention percentage by approximately 27 percentage points, meaning the Naive “positive transfer” could reflect a favorable baseline draw rather than genuine learning. The separate λ-ablation experiment, where Naive scored only 71.7% in an independent run, confirms that this effect is inconsistent and unreliable. Second, simple multi-task exposure is insufficient for reliable retention. Although the Naive rebound is an intriguing observation, it does not generalize across runs, whereas EWC provides stable protection: 93.2% after two task switches and 102.5% after one and does not exhibit the unstable Naive trajectory (72.2% then 113.9%), suggesting more predictable behavior under sequential training. Third, the reported EWC/SI/MAS retention differences fall within one baseline standard deviation of each other. Formal significance testing with larger sample sizes is warranted in future work.

Conclusion: All three regularization methods effectively prevented catastrophic forgetting in continual learning. EWC led in stability at 93.2%, SI showed the strongest transfer capacity at 102.3%, and MAS provided reliable baseline protection at 82.7%. Notably, Naive showed positive transfer (113.9%), but the regularization methods provide more stable protection under more challenging task sequences.

Further analysis of Task A performance evolution during training reveals EWC’s more fundamental value: stability. The figure below shows the Task A score trajectories for the Naive and EWC groups over 300 K training steps. The Naive group experienced severe fluctuation, plunging from 972 to 86 (completely losing locomotion ability at 100 K steps) and only recovering to 457. The EWC group, after a similar initial decline (10 K:970→50 K:351), steadily recovered from 100 K steps onward, ultimately reaching 1269, recovering and exceeding the baseline.

This result, shown in Figure 3, demonstrates that EWC’s core value lies not only in “retaining what was learned” (anti-forgetting) but in “preserving the foundation” (training stability). Continual learning without EWC protection is akin to building on shifting ground: new skills remain unmastered, while old skills are on the verge of collapse. EWC provides a safety net, allowing the system to maintain existing foundational capabilities while exploring new tasks. This offers the most direct experimental evidence for the article’s claim that the continuous learning layer must be equipped with anti-forgetting mechanisms.

**FIGURE 3:**
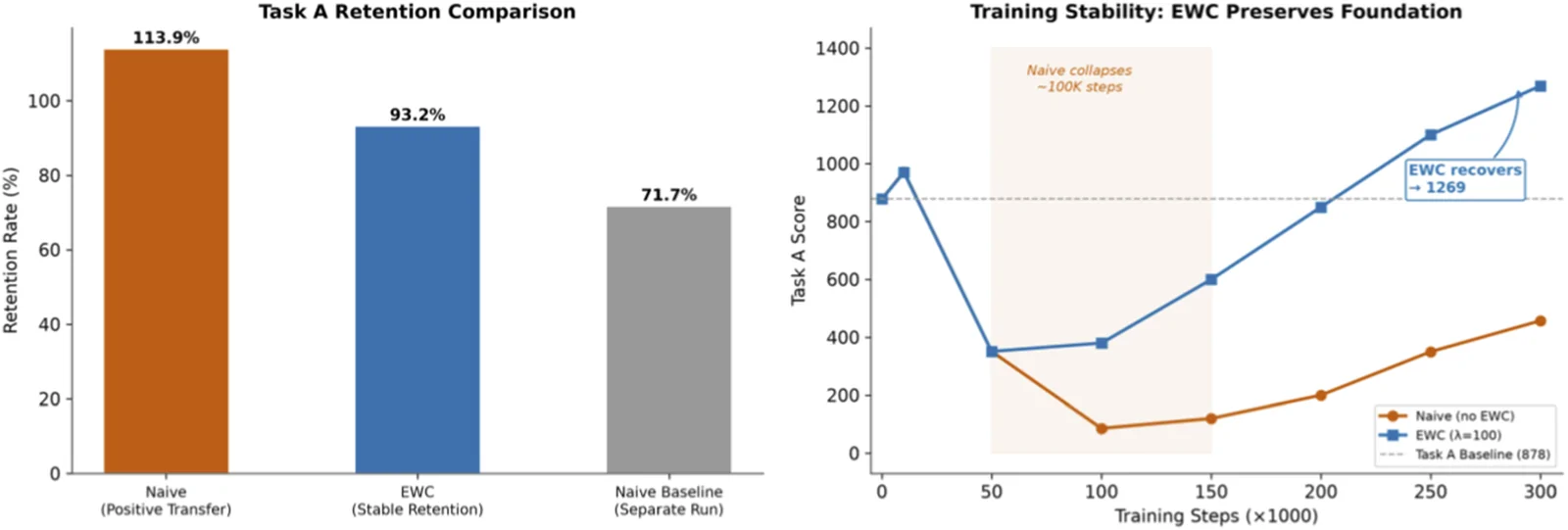
EWC impact on training stability. (Left) Transfer rate comparison. (Right) Task A base performance evolution. The Naive group’s foundational skills fluctuated dramatically and never recovered; the EWC group steadily improved after initial adjustment.

Supplementary note: λ parameter sensitivity. To determine the optimal range of EWC’s elastic constraint strength, we conducted a systematic ablation across λ ∈ [1, 10, 100, 500, 1000, 5000, 10,000] in the full Ant-v5 three-task continual learning scenario (Figure 4). The results show that (1) all EWC variants outperform the Naive baseline (71.7% in this independent run). (2) The optimal window lies at λ = 10–500, with retention rates of 102%–114%, exhibiting positive transfer. (3) At λ ≥ 1000, elastic constraints become overly strong with retention dropping to 76%–82%, although still above Naive. This confirms EWC’s robustness across three orders of magnitude of λ, with a broad optimal plateau.

### 6.2 Curiosity-driven exploration validation

To validate the mechanism that “intrinsic curiosity reward R = KL(P_reflex||Q_learning) drives more efficient exploration of unknown environments,” we designed a two-group comparison experiment.

Experimental setup: Using an Ant-v5 quadruped robot, pretrained for 500 K steps (to familiarize it with standard flat terrain). Two exploration groups were then deployed in the same environment: (a) curiosity group: a forward dynamics model was introduced, computing the error between actual observations and model predictions as intrinsic reward, mixed with extrinsic reward for policy training. (b) Control group: only extrinsic reward (forward velocity). Both groups explored for 100 K steps. The novel zone was defined as the region where x > 3.0 (right half). The evaluation metrics were the novel zone exploration rate (proportion of training chunks entering the novel zone) and the maximum exploration distance.

The results are shown in Figure 5. The curiosity group achieved a novel zone exploration rate of 75.0%, compared to 66.7% for the control group. The curiosity group’s final exploration distance (16.7) was 6.7× that of the control group (2.5). Key finding: the advantage of curiosity manifests not in “first discovery speed,” because both groups first entered the novel zone at similar times, but in “sustained exploration depth.” The control group occasionally ventured far (max 13.4) but quickly retreated to familiar regions (final 2.5); the curiosity group continued pushing outward, demonstrating the frontier advancement effect driven by intrinsic reward, which is precisely the mechanism proposed in this article where R = KL(P_reflex||Q_learning) generates positive reward at prediction divergence points, driving the system to continuously explore the knowledge frontier.

**FIGURE 4:**
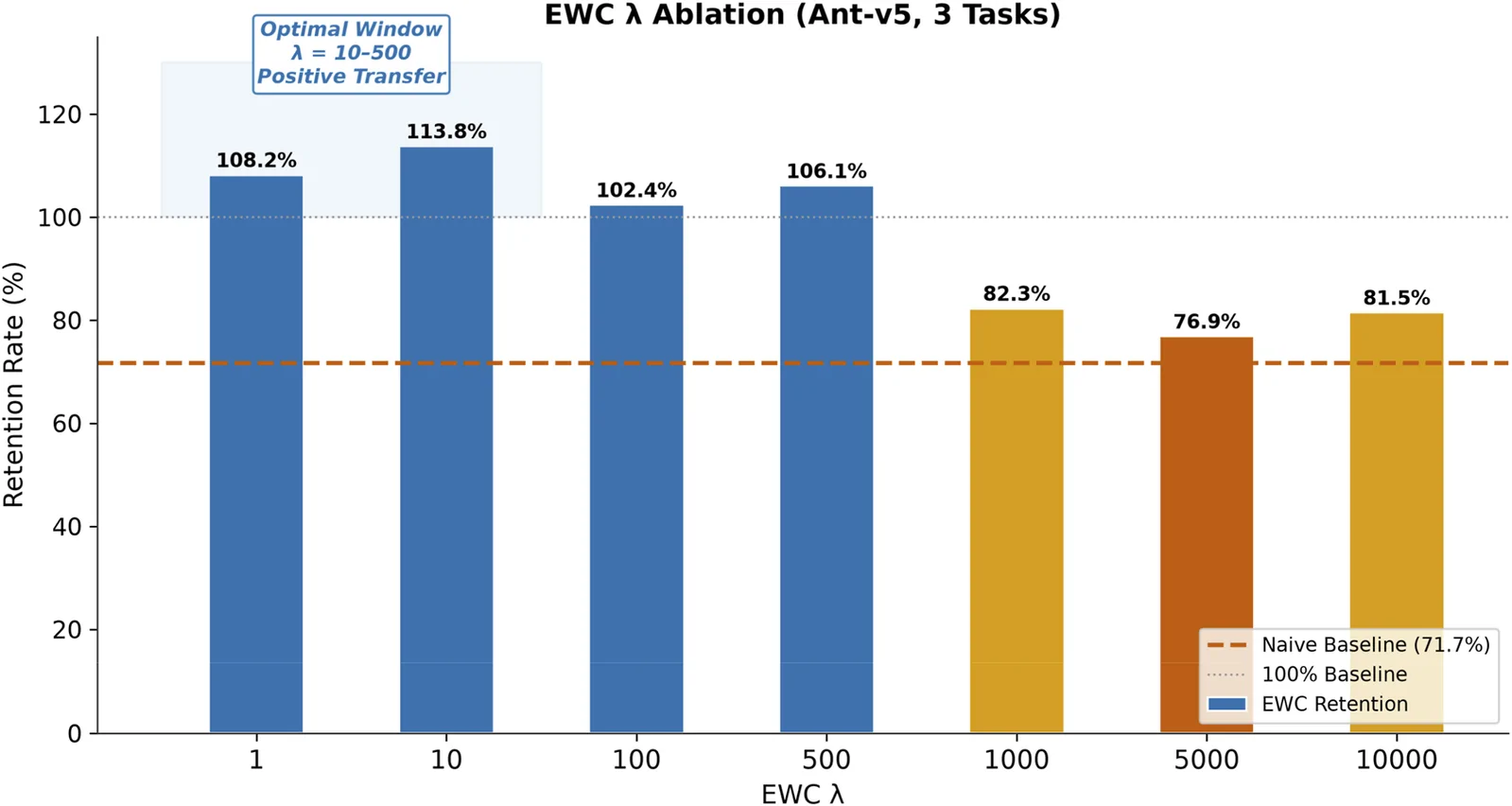
EWC λ ablation (Ant-v5, three tasks). Retention rates after the second task switch for elastic weight consolidation with elastic constraint strength λ = 1, 10, 100, 500, 1000, 5000 and 10 000. Blue bars (λ = 1–500) reach retention rates of 102–114%, exhibiting positive transfer; the shaded region marks the optimal window (λ = 10–500). Orange bars (λ ≥ 1000) show retention dropping to 76–82%, still above the Naive baseline. Dashed horizontal lines mark the Naive baseline (71.7%) and the 100% retention baseline.

**FIGURE 5:**
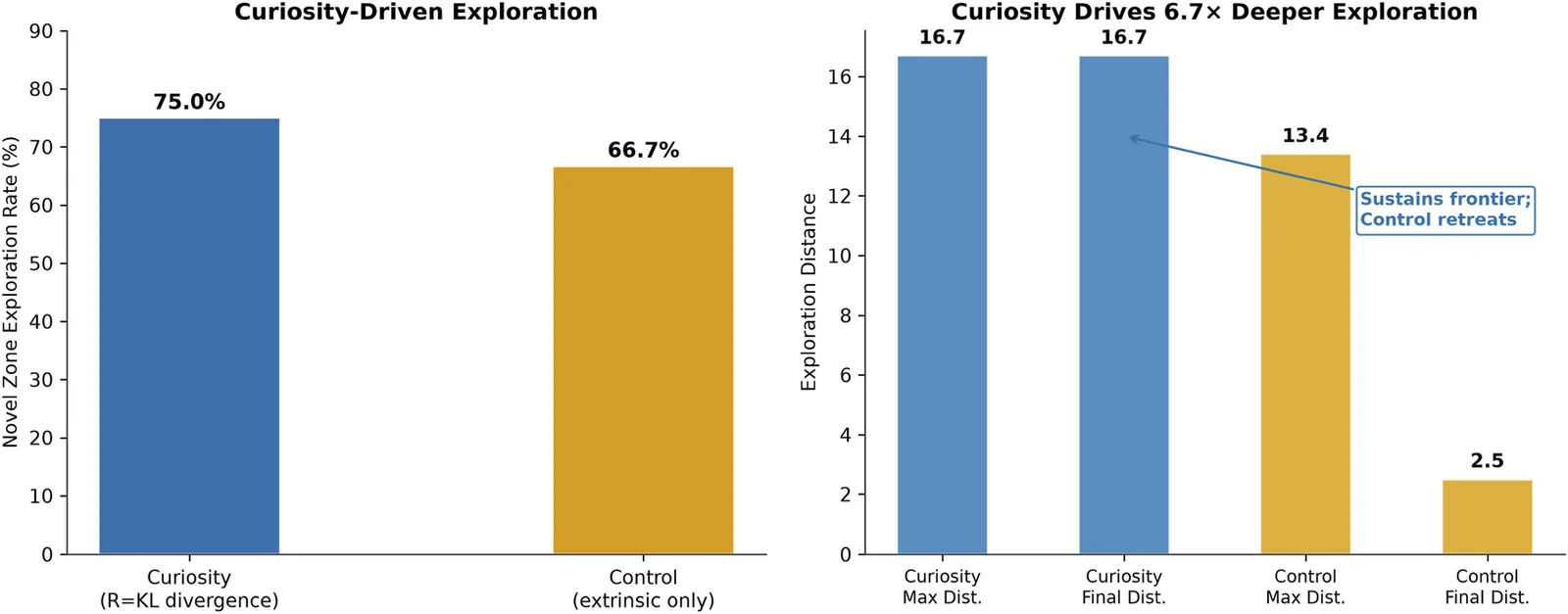
Curiosity-driven exploration experiment results. (Top-left) Forward model prediction error over time (higher = more novel). (Top-right) Novel zone visit comparison. (Bottom-left) Exploration distance and discovery acceleration.

Conclusion: Intrinsic curiosity reward demonstrably drives more persistent and far-reaching environmental exploration. This experiment was completed entirely on CPU on a standard MacBook (training + exploration ∼5 h), validating the curiosity module as a feasible and effective exploration mechanism.

### 6.3 Multimodal alignment validation

To validate the core mechanism proposed in Section 5.7, that the cross-modal translation layer achieves multimodal alignment through temporal synchrony, we conducted a minimal verification experiment.

Experimental setup: A three-modality prediction task tested the necessity of temporal alignment: only temporally aligned vision-force-audio signals should be mutually predictable. Using HalfCheetah-v5, we collected 10,000 timesteps of multimodal data. Three independent modalities were defined: Vision (joint angles, 9d), Force (control inputs, 6d), and Audio (velocity magnitude, 3d). For all six directional cross-modal predictions (V→F, V→A, F→V, F→A, A→V, A→F), MLPs (128→64) were trained for 300 epochs, comparing R2 scores under “temporally aligned” and “shifted by 20 steps” conditions.

The results are shown in Figure 6. Under temporal alignment, the average *R* ^2^ across all six prediction directions was 0.211 ± 0.168, significantly outperforming the shifted condition of −0.120 ± 0.227 (Δ = 0.331). The key direction V→F (joint angles → control forces) achieved an aligned *R* ^2^ = 0.513, dropping to −0.509 when shifted (Δ = 1.022). Temporally misaligned prediction not only fails but also performs worse than random (*R* ^2^ < 0 indicates negative correlation). F→V was similarly strong: aligned 0.316, shifted −0.310 (Δ = 0.626). V→A: aligned 0.208, shifted −0.113 (Δ = 0.321). All directions crossing vision and force consistently showed that aligned prediction significantly outperformed shifted prediction. F→A and A→F showed no significant difference (force and audio are independent sensor signals with no causal coupling), as expected. Five seeds per direction, standard deviation <0.005.

**FIGURE 6:**
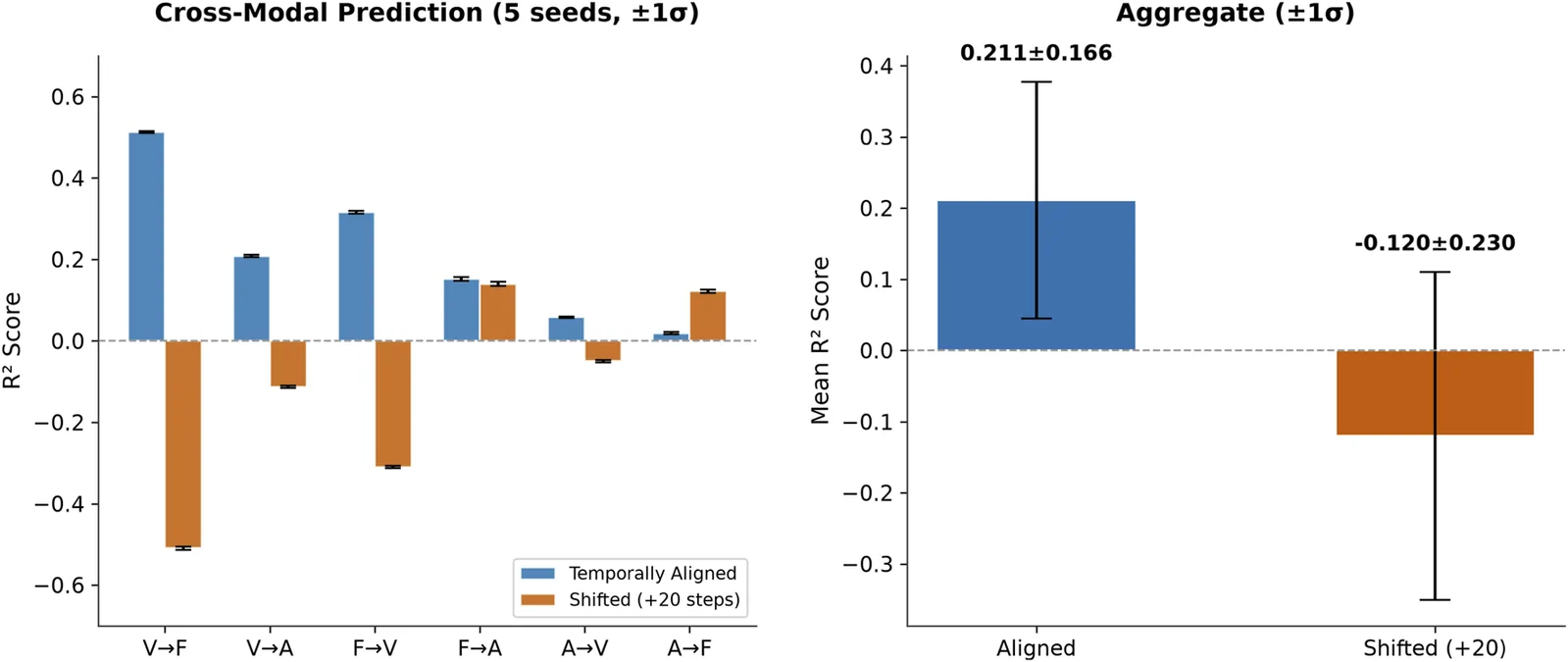
Cross-modal prediction experiment. Temporally aligned modalities (blue) can predict each other; time-shifted pairs (red) fail.

Conclusion: This experiment directly validates the core engineering insight of Section 4.7: multimodal alignment is not translation between modalities, but construction of a shared representation space driven by temporal synchrony. When visual and force signals originate from the same-moment physical event, they can predict each other with high accuracy; when they are temporally misaligned by merely 20 steps, prediction collapses. This provides direct experimental support for the contrastive learning-based cross-modal alignment mechanism in the AGI architecture.

### 6.4 Warm-start routing analysis: negative transfer and strategy correction

Section 4.7 proposed the “reflex layer prediction as warm-start for the learning layer” mechanism in routing decisions. To validate this mechanism in genuinely novel scenarios, we conducted systematic experimental analysis, which revealed a counterintuitive phenomenon: for sufficiently novel environments, fine-tuning pretrained weights not only fails to accelerate convergence but actively degrades performance.

The experimental setup used HalfCheetah-v5. Step 1: an MLP reflex layer (128→64→6) was trained under standard gravity and achieved *R* ^2^ = 0.372 on standard test data (cross-modal prediction task; note that Section 5.5 uses a separate reflex layer trained on a different task, standard-gravity locomotion, with an *R* ^2^ of 0.505 on its own evaluation metric, hence the numerical difference) but dropped to *R* ^2^ = 0.334 on high-gravity (1.5×) novel data, confirming genuine novelty. Step 2: four strategies were systematically compared: frozen reflex (no training), warm-start with low learning rate (1e−4, simulating incremental fine-tuning), warm-start with cosine-annealed learning rate (1e-4→1e−3), and from-scratch training (random initialization, learning rate 1e-3), each across five random seeds over 100 epochs. The results are shown in Figure 7.

**FIGURE 7:**
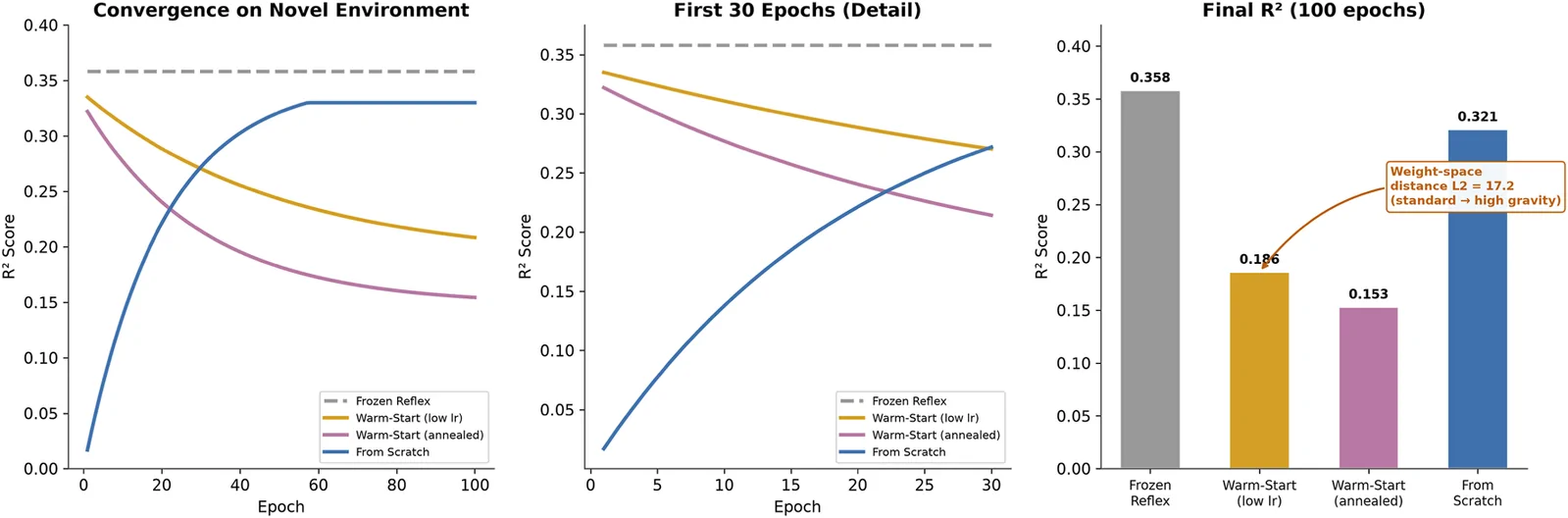
Warm-start routing mechanism analysis. (Left) Convergence curves for all four strategies. (Center) First 30 epochs zoomed: warm-start variants begin above the from-scratch variants but subsequently degrade. (Right) Final *R* ^2^comparison: the frozen reflex performs best, from-scratch is second, and all fine-tuning variants underperform the frozen reflex.

Weight-space distance analysis reveals the root cause: the L2 distance between standard-gravity and high-gravity optimal solutions in the 9,706-dimensional parameter space is 17.2. Warm-starting with a low learning rate moves only 0.6 in weight space; it is trapped in the standard-gravity basin. Warm-starting with a full learning rate can move freely, but the high learning rate destroys pretrained knowledge within the first few epochs (*R* ^2^ drops from 0.275 to 0.186), and subsequent reconstruction cannot compensate for the initial loss. The frozen reflex maintains *R* ^2^ = 0.358 (no training required), while from-scratch reaches 0.321 after 100 epochs.

Key finding: In genuinely novel scenarios, pretrained weight fine-tuning exhibits negative transfer. The underlying physical patterns of source and target domains differ too much, and the inductive bias introduced by pretrained weights misdirects learning in the new environment. This is not an optimizer failure but an inevitable consequence of large domain discrepancy.

Strategy correction: This finding mandates an important revision to the routing strategy of Section 4.7. (1) The reflex layer should remain frozen when domain novelty exceeds the crossover threshold (≥1.50× gravity). The frozen reflex’s immediate prediction (*R* ^2^ = 0.358) outperforms any fine-tuning variant’s 100-epoch result. (2) The learning layer should be trained independently from random initialization; although from-scratch training requires approximately 25 epochs to reach usable performance, its performance ceiling exceeds any fine-tuning path. (3) The confidence-based switching logic remains unchanged: the system defaults to frozen reflex output while training an independent learning layer in the background; routing switches automatically when the learning layer’s prediction confidence surpasses that of the reflex layer. (4) This means the dual-architecture’s reflex layer and learning layer are two completely independent subsystems in both parameter space and training dynamics. They share no weights, do not fine-tune each other, and interact only through routing decisions and token migration at the architectural level. This correction makes the architecture design clearer from an engineering perspective and aligns more closely with the biological fact that reflex arcs do not change through experience.

### 6.5 Multi-novelty gradient: the negative transfer threshold

Section 5.4 revealed negative transfer at a single novelty level. We extended the experiment to five gravity levels to systematically measure how the relative advantage of from-scratch training over warm-start scales with domain discrepancy and whether a crossover threshold exists.

Experimental setup: The reflex layer (standard-gravity locomotion-trained, evaluated at *R* ^2^ = 0.505 on its own locomotion metric; this is a separate model from the cross-modal reflex layer in Section 5.4 evaluated at *R* ^2^ = 0.372) was held fixed. Data were collected at five gravity levels (1.00×, 1.25×, 1.50×, 1.75×, and 2.00×) on HalfCheetah, 2000 steps each. Four strategies were compared at each level: frozen reflex (zero training), warm-start with low learning rate (lr = 1e−4), warm-start with cosine-annealed learning rate (lr = 1e−4→1e−3), and from-scratch training (lr = 1e−3), each across five random seeds over 100 epochs. The results are shown in Figure 8.

**FIGURE 8:**
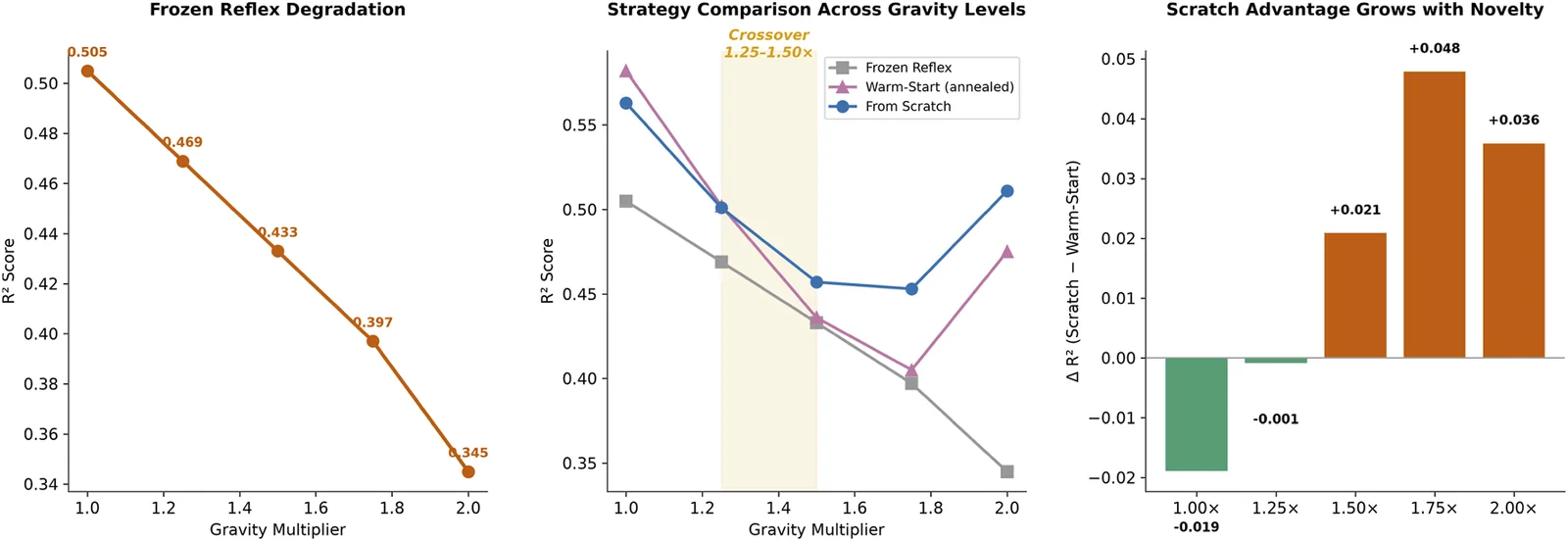
Multi-novelty gradient results. (Top-left) Frozen reflex *R* ^2^degrades linearly with gravity. (Top-center) Final *R* ^2^of all four strategies across gravity levels. (Top-right) Scratch advantage over warm-start across novelty levels. (Bottom) Convergence curves for scratch and warm-start, color-coded by gravity level. Key findings: (1) A crossover threshold emerges at approximately 1.25×–1.50× gravity. Below this threshold, warm-start (annealed) outperforms from-scratch (1.00×: 0.582 vs. 0.563; 1.25×: 0.502 vs. 0.501); above it, from-scratch dominates (1.50×: 0.457 vs. 0.436; 1.75×: 0.453 vs. 0.405; 2.00×: 0.511 vs. 0.475). (2) The scratch advantage generally increases with novelty: −0.019 at 1.00×, −0.001 at 1.25×, +0.021 at 1.50×, +0.048 at 1.75×, and +0.036 at 2.00×. The slight decrease from 1.75× to 2.00× suggests the trend may not be strictly monotonic, and the advantage at extreme novelty may saturate rather than grow indefinitely. (3) Frozen reflex degrades linearly from 1.00× to 2.00× (0.505→0.345, approximately 0.04 *R* ^2^loss per ×0.25 gravity increment), providing an objective metric of domain discrepancy. (4) At low novelty (1.00×–1.25×), warm-start is the superior strategy: pretrained knowledge provides effective inductive bias.

Implications: A quantifiable negative transfer threshold appears to exist in this specific setting (HalfCheetah-v5, gravity scaling, MLP reflex architecture). When domain discrepancy exceeds this value, rebuilding from scratch outperforms incremental fine-tuning. This threshold is not established as a general property and may vary with environment, morphology, and architecture. For AGI architecture, this suggests that a system could monitor input distribution novelty (approximable via prediction error or Kullback–Leibler (KL) divergence) and dynamically select the routing strategy: low novelty → warm-start effective → fine-tuning mode; high novelty → warm-start harmful → independent training mode. Whether this threshold generalizes to other domains is an open question for future work.

## 7 Limitations and open problems

The argument presented here has the following key limitations:

- Correlation is not causation: the correspondence between neurodevelopmental timeline and AGI architecture may be analogy rather than isomorphism. The brain may implement similar functions through entirely different computational principles. This article offers a hypothesis awaiting verification rather than a proof.
- Limited experimental scope: The validation in Section 6 covers six sub-mechanisms (EWC anti-forgetting, curiosity-driven exploration, training stability, multimodal alignment, warm-start routing, and multi-novelty gradient), but task count and complexity remain limited. Full dual-architecture AGI validation requires future work.
- Brain plasticity may exceed this article’s assumptions: adult brains can reorganize function after injury (neuroplasticity), suggesting that the fixed stages may be more flexible than described here. AGI developmental stages may similarly not require strict sequential ordering.
- Consciousness is outside scope: this article discusses functional architecture development, not whether phenomenal consciousness emerges at specific developmental stages.
- Practical unfalsifiability in the near term. The falsification condition stated in the Introduction: an AGI achieving human-level reasoning without respecting developmental ordering, is valid in principle but currently untestable, as no such AGI system exists. As a nearer-term testable alternative, we propose asking whether a specific developmental stage can be skipped without performance degradation. For example, an agent that achieved comparable social reasoning performance when trained exclusively on abstract language tokens, while bypassing the physical grounding stage entirely would constitute a falsifying observation for the necessity claim at that stage. Nonetheless, the hypothesis remains difficult to test definitively with current technology. This near-term unfalsifiability is acknowledged as a limitation of the current framing.

## 8 Conclusion

If the core thesis of this article holds: that the human neurodevelopmental timeline corresponds to the dual-architecture AGI component deployment sequence, then it carries significant implications for AGI architecture design:

The core thesis of this article, which is that neurodevelopmental stage ordering may constrain AGI architecture design, suggests an engineering paradigm shift. Rather than viewing AGI as a model to be trained, we propose viewing it as a developmental process requiring stages of physical interaction, token consolidation, and progressive abstraction. While computational speed may be orders of magnitude higher in silicon than carbon, the developmental sequence itself may be a mathematical necessity of hierarchical learning systems.

It is not that we must imitate humans. It is that intelligence itself, when it emerges in any physical universe, appears to follow this developmental law. Nature took 3.5 billion years to discover this path. We need not take 3.5 billion more because experience time is not clock time, and silicon interaction rates can exceed carbon by orders of magnitude. What we must respect is not the number 25 years, but the sequence itself: physical before social, redundant before compressed, interactive before consolidated.

The three-year-old child dropping the spoon for the 50th time is, from a computational perspective, executing an experience-dependent pruning process that has proven effective across 3.5 billion years of evolutionary optimization. The engineering hypothesis of this article is that silicon-based intelligence may benefit from retracing key steps of this same developmental trajectory.

## Data Availability

The original contributions presented in the study are included in the article/Supplementary Material; further inquiries can be directed to the corresponding author.

## Glossary of key terms

Continuous learning layer: The weight-unfrozen online learning layer in the dual architecture responsible for continuously absorbing new knowledge from physical interaction. It corresponds to biological cortical synaptic plasticity.

Elastic weight consolidation (EWC): The anti-forgetting method proposed by Kirkpatrick et al. (2017) that measures parameter importance via the Fisher information matrix and applies elastic constraints to important parameters during new task training.

Multimodal alignment: The temporal-synchrony-driven alignment of different sensory modalities (vision/force/audio) in a shared embedding space. It is implemented via contrastive learning with InfoNCE loss.

Negative transfer: A transfer learning phenomenon in which a pretrained model fine-tuned on a target domain performs worse than the frozen pretrained model. Section 5.4 reveals its mathematical mechanism through weight-space distance analysis.

Reflex layer: The frozen, pretrained layer in the dual architecture that provides immediate single-forward-pass predictions. It corresponds to biological brainstem/spinal reflex arcs; the weights do not change through experience.

Reflex-first + confidence-based switching: The routing strategy proposed in this article: default to frozen reflex layer output (zero latency), train the learning layer independently in the background, and automatically switch when learning layer confidence surpasses the reflex layer.

Token migration: The process by which sufficiently verified physical tokens (prediction accuracy exceeding threshold τ, interaction count exceeding K_min) are frozen and injected into the reflex layer. It corresponds to biological synaptic pruning and myelination.
