# Supplementary material for: Neurodevelopment as blueprint: biological validation of the dual-architecture AGI framework
Source: Front Robot AI. 2026 Oct 8;13:1902952. doi: 10.3389/frobt.2026.1902952 (PMC13686898; doi:10.3389/frobt.2026.1902952)
Supplement: Supplementary file 1 [file Supplementaryfile1.pdf]

Appendix: Formal Theory of Token Migration

This appendix formalizes the physical-to-social token migration process introduced in Section 2.3, proves that migration fidelity is positively correlated with the Fisher information of the source token, and derives an upper bound on transfer loss.

A.1 Definitions

Let the physical token space be $P \subseteq R^{d_p}$, where each physical token's vector encoding $v_p \in P$ is obtained through physical interaction verification. Let the social token space be $S \subseteq R^{d_s}$, where social token encodings $v_s \in S$ are not yet acquired. The domain transfer matrix $W \in R^{d_s \times d_p}$ maps physical space to social space. The transferred social token candidate is $\hat{v}_s = W \cdot v_p$.

Define the Fisher information of a token: For physical token p, the average diagonal Fisher information is $F(p) = (1/d_p) \Sigma_i F_{ii}$, where $F = E[(\nabla_\theta \log p(O|\theta))^2]$ is the Fisher information matrix estimated at the optimal parameters θ^*. Fisher information measures how thoroughly a token has been verified through physical interaction — higher $F(p)$ means a more stable token.

Define migration fidelity: The target vector for social token s is v^*_s (the ideal encoding obtained after sufficient social interaction). Migration fidelity is the cosine similarity between the transferred vector and the target: $\varphi(p, s) = \cos(\hat{v}_s, v^*_s) = (\hat{v}_s \cdot v^*_s) / (\|\hat{v}_s\| \cdot \|v^*_s\|)$.

Formal Sketch 1: Fisher Information Positively Correlates with Fidelity

Formal Sketch 1: Let the Fisher information of physical token p be $F(p)$, and the transferred social token candidate be $\hat{v}_s = W \cdot v_p$. Then migration fidelity $\varphi(p, s)$ is positively correlated with $F(p)$. That is, for two physical tokens p_1 and p_2, if $F(p_1) > F(p_2)$, then $E[\varphi(p_1, s)] \geq E[\varphi(p_2, s)]$.

Derivation: The vector encoding v_p of a physical token is obtained by minimizing prediction error. Near the optimal parameters θ^*, the covariance matrix of v_p approximates the inverse Fisher information matrix: $Cov(v_p) \approx F^{-1}(p)$. Therefore, larger $F(p)$ implies smaller variance — the token's vector representation is more precise. The target social token $v^*_s = W \cdot v^*_p + \varepsilon$, where ε is the residual due to physical-social structural differences. As $v_p \to v^*_p$ (high Fisher information), migration fidelity $\varphi \to 1 - O(\|\varepsilon\|)$. Hence, higher $F(p)$ yields higher fidelity.

A.3 Transfer Loss Upper Bound

Formal Sketch 2: The expected domain transfer loss is bounded by $E[L_{transfer}] \leq \|W\|_F \cdot (Tr(F^{-1}(p))/d_p)^{1/2} + \|\varepsilon\|$, where $\|W\|_F$ is the Frobenius norm of the transfer matrix, and ε is the irreducible structural difference between physical and social domains.

Derivation: Transfer loss $L_{transfer} = \|W \cdot v_p - (W \cdot v^*_p + \varepsilon)\|^2 = \|W(v_p - v^*_p)\|^2 + \|\varepsilon\|^2 + 2(W(v_p - v^*_p)) \cdot \varepsilon$. Taking expectations, since $E[v_p - v^*_p] = 0$ and the cross-term expectation is zero, $E[L_{transfer}] = E[\|W(v_p - v^*_p)\|^2] + \|\varepsilon\|^2$. By Cauchy-Schwarz, $E[\|W(v_p - v^*_p)\|^2] \leq \|W\|_F^2 \cdot E[\|v_p - v^*_p\|^2]$. And $E[\|v_p - v^*_p\|^2] = Tr(Cov(v_p)) \approx Tr(F^{-1}(p))/d_p$. Therefore $E[L_{transfer}] \leq \|W\|_F^2 \cdot Tr(F^{-1}(p))/d_p + \|\varepsilon\|^2$. Taking the square root yields the bound.

The engineering implication of this theorem: To reduce transfer loss, one can (1) increase the Fisher information of physical tokens (more interaction verification) — this is the theoretical basis for the τ threshold and K_{min} minimum interaction count in the paper; (2) optimize the norm of the transfer matrix W (learn a better cross-domain mapping); (3) reduce ε — but ε represents essential structural differences between physical and social domains, which may be irreducible.

A.4 Corollary: Developmental Necessity of Migration Order

Corollary 1: If $F(p) < F_{min}$ (the physical token has not reached the minimum verification threshold), then the expected lower bound of migration fidelity falls below random level, i.e., $E[\varphi(p, s)] \leq \varphi_{random}$. In this case, migration cannot produce a meaningful social token — the system must wait for the physical token to accumulate sufficient Fisher information through interaction.

This provides a mathematical explanation for the paper's core claim: social tokens cannot be acquired before physical tokens, not because "social is harder," but because the vector encoding of a social token mathematically depends on the vector encoding of a physical token as a prior. Before the transfer matrix W is learned, any attempt to learn v_s directly from social interaction is equivalent to random search in an ungrounded feature space.
